# Performance of Machine Learning Algorithms for Predicting Progression to Dementia in Memory Clinic Patients

**DOI:** 10.1001/jamanetworkopen.2021.36553

**Published:** 2021-12-16

**Authors:** Charlotte James, Janice M. Ranson, Richard Everson, David J. Llewellyn

**Affiliations:** 1 University of Exeter Medical School, Exeter, United Kingdom; 2 Deep Dementia Phenotyping Network, United Kingdom; 3 Department of Computer Science, University of Exeter, Exeter, United Kingdom; 4 The Alan Turing Institute, London, United Kingdom

## Abstract

**Importance:**

Machine learning algorithms could be used as the basis for clinical decision-making aids to enhance clinical practice.

**Objective:**

To assess the ability of machine learning algorithms to predict dementia incidence within 2 years compared with existing models and determine the optimal analytic approach and number of variables required.

**Design, Setting, and Participants:**

This prognostic study used data from a prospective cohort of 15 307 participants without dementia at baseline to perform a secondary analysis of factors that could be used to predict dementia incidence. Participants attended National Alzheimer Coordinating Center memory clinics across the United States between 2005 and 2015. Analyses were conducted from March to May 2021.

**Exposures:**

258 variables spanning domains of dementia-related clinical measures and risk factors.

**Main Outcomes and Measures:**

The main outcome was incident all-cause dementia diagnosed within 2 years of baseline assessment.

**Results:**

In a sample of 15 307 participants (mean [SD] age, 72.3 [9.8] years; 9129 [60%] women and 6178 [40%] men) without dementia at baseline, 1568 (10%) received a diagnosis of dementia within 2 years of their initial assessment. Compared with 2 existing models for dementia risk prediction (ie, Cardiovascular Risk Factors, Aging, and Incidence of Dementia Risk Score, and the Brief Dementia Screening Indicator), machine learning algorithms were superior in predicting incident all-cause dementia within 2 years. The gradient-boosted trees algorithm had a mean (SD) overall accuracy of 92% (1%), sensitivity of 0.45 (0.05), specificity of 0.97 (0.01), and area under the curve of 0.92 (0.01) using all 258 variables. Analysis of variable importance showed that only 6 variables were required for machine learning algorithms to achieve an accuracy of 91% and area under the curve of at least 0.89. Machine learning algorithms also identified up to 84% of participants who received an initial dementia diagnosis that was subsequently reversed to mild cognitive impairment or cognitively unimpaired, suggesting possible misdiagnosis.

**Conclusions and Relevance:**

These findings suggest that machine learning algorithms could accurately predict incident dementia within 2 years in patients receiving care at memory clinics using only 6 variables. These findings could be used to inform the development and validation of decision-making aids in memory clinics.

## Key Points

**Question:**

Can machine learning algorithms accurately predict 2-year dementia incidence in memory clinic patients and how do these predictions compare with existing models?

**Findings:**

In this prognostic study of data from 15 307 memory clinic attendees without dementia, machine learning algorithms were superior in their ability to predict incident dementia within 2 years compared with 2 existing predictive models. Machine learning algorithms required only 6 variables to reach an accuracy of at least 90%, and had an area under the receiver operating characteristic curve of 0.89.

**Meaning:**

These findings suggest that machine learning algorithms could be used to accurately predict 2-year dementia risk and may form the basis for a clinical decision-making aid.

## Teaser

This prognostic study assesses the ability of novel machine learning algorithms compared with existing risk prediction models to predict dementia incidence within 2 years.

## Introduction

Many patients assessed in specialist settings, such as memory clinics, do not have dementia when they first attend.^1^ Differentiating between patients who go on to develop dementia within a clinically relevant timeframe and those who remain dementia-free is important, as that insight can be used to prioritize patients for follow-up investigations and interventions. Identifying patients at high risk of developing dementia is challenging for clinicians. One approach is to focus on those who have mild cognitive impairment (MCI) when initially assessed and invite these patients for follow-up. However, this can result in considerable misclassification for patients who are not targeted for follow-up but who develop dementia and patients who are targeted for further investigations but do not develop dementia. Most memory clinic patients with MCI do not progress to dementia even after 10 years, with an annual conversion rate of 9.6%.^2^

Clinical decision-making aids may improve the ability of clinicians to estimate dementia onset. Existing clinical decision-making aids are available to estimate medium- and long-term incidence of dementia in different populations. For example, the Cardiovascular Risk Factors, Aging, and Incidence of Dementia (CAIDE) Risk Score^3^ was designed to predict risk for developing dementia in 20 years for middle-aged people, and the Brief Dementia Screening Indicator (BDSI)^4^ aims to identify elderly patients to target for cognitive screening by determining their risk of developing dementia in 6 years. However, to our knowledge, no clinical decision-making aid has been developed to predict dementia incidence in memory clinics over a shorter clinically relevant period.

Machine learning (ML) allows for the leverage of information from large and complex data sets. Recently, it has been applied to dementia diagnosis and risk prediction.^5,6,7,8,9^ However, these models often incorporate information not typically available in routine clinical practice, such as advanced neuroimaging, genetic testing, and cerebrospinal fluid biomarkers, limiting clinical application to specialist or research settings.

We investigated whether ML techniques can be used to predict the incidence of dementia over a 2-year period using memory clinic data from the US National Alzheimer Coordinating Center (NACC). We also examined the minimum set of variables required for ML models to reach full diagnostic performance.

## Methods

The NACC study received ethical approval from each site’s institutional review board before it could contribute data, and all participants had provided informed written consent. This prognostic study was deemed exempt from institutional ethical approval because we used previously collected deidentified data. The data used in this study are available by data request to the NACC. This study is reported in accordance with the Transparent Reporting of a Multivariable Prediction Model for Individual Prognosis or Diagnosis (TRIPOD) reporting guideline. Data were analyzed from March to May 2021.

### Study Sample

We used previously collected data from the NACC Uniform Data Set (UDS).^10^ The UDS contains prospective cohort data from the US National Institute on Aging Alzheimer Disease Center program for multicenter collaborative research on Alzheimer disease and other neurodegenerative disorders.^11^ Our data set consists of memory clinic data collected between September 2005 and February 2015 from 30 Alzheimer Disease Centers located in the United States. The data set includes participant and coparticipant sociodemographic characteristics, family history, functional status,^12^ behavioral symptoms (assessed with Neuropsychiatric Inventory Questionnaire results^13^), neuropsychological test battery,^14^ and NACC clinical dementia diagnosis, assigned by each Alzheimer Disease Center using published clinical diagnostic criteria based on the standardized UDS clinical evaluation. Details of the diagnostic criteria adopted by the UDS protocol and the associated guidance have been published previously.^15^

We used UDS versions 1 and 2, which include 32 573 memory clinic attendees with a baseline assessment. Although our models are designed to predict dementia incidence within 2 years, to account for variation in the time between follow-up appointments, we included follow-up that occurred within 29 months of the initial visit to ensure that the visit was either the first or second follow-up appointment.

### Outcome Variable

The outcome variable was incident all-cause dementia diagnosis within 29 months (approximately 2 years) of baseline assessment. This includes dementia subtypes, such as Alzheimer dementia, dementia with Lewy bodies, vascular dementia, and other rarer subtypes. Alzheimer dementia was diagnosed according to NINCSD-ADRDA criteria,^16^ vascular dementia was diagnosed according to NINDS-AIREN criteria,^17^ Lewy body dementia (LBD) was diagnosed according to the third report of the Dementia with Lewy Bodies Consortium criteria,^18^ and frontotemporal dementia was diagnosed according to Neary and colleagues’ 1998 criteria.^19^

### Candidate Predictors

We included all clinically relevant variables collected during the initial visit in versions 1 and 2 of the UDS (eTable 1 in the Supplement). We excluded variables with free text values, such as names of medications, and variables that were constant across all participants, such as visit number. Four synthetic variables were generated to help with the evaluation of variable importance (these variables should be ranked low); 3 of these variables were permutations of existing variables (1 binary, 1 categorical, and 1 numerical variable), and 1 variable was randomly generated from a normal distribution. This resulted in a total of 258 variables.

The variables from the UDS incorporated into our models include participant demographic characteristics (15 variables), coparticipant demographic characteristics (7 variables), family history (3 variables), medical history (47 variables), medications (21 variables), physical (12 variables) and neurological (4 variables) examination results, the Unified Parkinson Disease Rating Scale^20^ (UDPRS) (28 variables), Clinical Dementia Rating (CDR) scale^21^ (8 variables), functional status (10 variables), neuropsychological test battery (50 variables), Geriatric Depression Scale (17 variables), and a clinical assessment of symptoms (32 variables). Of these variables, 239 (93%) were missing for at least 1 participant, and all participants had at least 1 variable missing.

### Model Development

We implemented 4 ML algorithms^22^: logistic regression (LR),^23^ support vector machine (SVM),^24^ random forest (RF),^25,26^ and gradient-boosted trees (XGB)^27^ (eMethods in the Supplement). These algorithms perform a classification task: they determine whether a participant falls into class 0 (predicted to remain dementia-free 29 months from baseline) or class 1 (predicted to experience incident dementia within 29 months of baseline). The classification is based on variables recorded at their first (baseline) memory clinic visit. To implement the ML algorithms, we used the Python scikit-learn library (Python Software Foundation),^28^ with 5-fold cross validation (eMethods in the Supplement). Missing values were imputed by sampling with replacement from nonmissing values. All data processing and analysis was implemented in Python version 3.9, NumPy version 1.19.4, and scikit-learn version 0.24.0.

### Statistical Analysis

#### Model Evaluation

We evaluated the performance of all models by comparing their overall accuracy, sensitivity, and specificity for decision thresholds prespecified in the literature (existing models) or a threshold of 0.5 (ML models), which equally weights false-positive and false-negative errors. Area under the receiver operating characteristic curve (AUC)^29^ was used to summarize model performance over all possible thresholds and thus misclassification error weightings.^30^ Mean performance measures and SDs were obtained through bootstrapping (eMethods in the Supplement).

#### Comparison With Existing Models

The BDSI and CAIDE are existing dementia risk prediction models that assign to patients a score representing their risk of developing dementia over longer timescales. To derive the BDSI and CAIDE risk scores, we selected variables from the UDS that most closely correspond to variables used previously (eTable 2 in the Supplement). The performance of our ML models was compared with that of the BDSI and CAIDE for predicting 2-year dementia incidence.

#### Model Performance Across Dementia Subtypes

Dementia can have a variety of causes, corresponding to different dementia subtypes. To assess the ability of the ML models to identify different dementia subtypes, we divided the incident dementia cases into Alzheimer dementia, LBD, vascular dementia, and other dementia subtypes. Using these 4 stratifications, we calculated the percentage of participants correctly classified (true-positive rate) and compared the ROC curves for each ML model.

#### Investigation of Diagnostic Stability

The clinical diagnosis of dementia is known to incorporate patients who are initially misdiagnosed (effectively both false-positive and false-negative errors).^31^ We define *reversion* as when a participant who was diagnosed with dementia up to 2 years after their first memory clinic visit and subsequently receives a diagnosis of no dementia (either MCI or unimpaired cognition) within 2 years of their dementia diagnosis. Reasoning that these reversions are unstable diagnoses and likely to have been the result of dementia misdiagnosis, we investigated the classification accuracy of ML models in a sample of participants with reversion (eMethods in the Supplement). We used the cumulative distribution function (CDF) of classification scores output by each ML model to compare participants with reversion with patients who did develop dementia and patients who remained dementia free.

## Results

**Figure 1.**
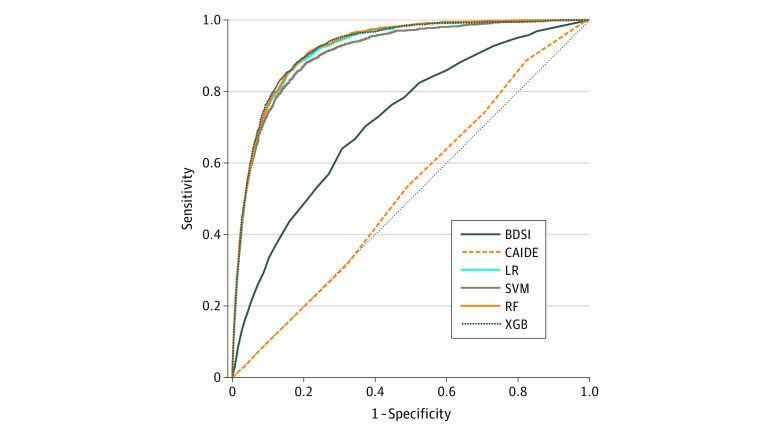
Receiver Operating Characteristic Curves BDSI indicates Brief Dementia Screening Indicator; CAIDE, Cardiovascular Risk Factors, Aging and Incidence of Dementia; LR, logistic regression; RF, random forest; SVM, support vector machine; and XGB, gradient-boosted trees.

After excluding 12 136 attendees with a diagnosis of dementia at baseline, 4557 attendees who did not have any follow-up data, and 573 attendees who had their first follow-up more than 29 months after their first visit, the final analytic sample contained 15 307 participants (mean [SD] age, 72.3 [9.8] years; 9129 [60%] women and 6178 [40%] men). Sample characteristics are shown in Table 1. Within 2 years of baseline, 1568 participants (10%) received a diagnosis of dementia. Of 1568 participants who received a diagnosis of dementia, 273 (17%) were diagnosed by a single clinician and 1216 (78%) were diagnosed by a consensus panel; for 79 participants (5%), the source of diagnosis was not specified. Key performance measures assessing the predictive power of each model are given in Table 2. Compared with existing models, ML models were superior in their ability to predict whether an individual would develop dementia within 2 years, and they outperformed existing models on all measures. All ML models performed similarly well, with XGB having the greatest power when measured by overall accuracy (92%) and AUC (mean [SD], 0.92 [0.01]). The receiver operating characteristic curve for each model demonstrates the similarity among the ML models and their superiority compared with the 2 existing risk models (Figure 1).

**Table 1.** Sample Characteristics

Characteristic	Participants, No. (%)	
	No incident dementia (n = 13 379)	Incident dementia (n = 1568)
Age, mean (SD), y	72 (9.8)	75 (9.4)
Sex		
Men	5376 (39)	802 (51)
Women	8363 (61)	766 (49)
Native English speaker	12 823 (93)	1471 (94)
Education, mean (SD), y	15.5 (3.2)	15.3 (3.3)
Dependent living	927 (7)	625 (40)
CDR sum, median (IQR)	0.0 (0.0-0.5)	1.5 (1.0-2.5)
Total MMSE score, mean (SD)	28.5 (1.8)	26.2 (2.7)

Abbreviations: CDR, Clinical Dementia Rating; MMSE, Mini-Mental State Examination.

**Table 2.** Performance Measures for the Prediction of Incident All-Cause Dementia Over 2 Years

Performance measures	Mean (SD)					
	Existing models^a^		Machine learning models^b^			
	BDSI	CAIDE	LR	SVM	RF	XGB
Overall accuracy	0.83 (0.01)	0.76 (0.01)	0.92 (0.01)	0.92 (0.01)	0.92 (0.01)	0.92 (0.01)
Sensitivity	0.37 (0.03)	0.18 (0.02)	0.47 (0.05)	0.47 (0.05)	0.31 (0.05)	0.45 (0.05)
Specificity	0.88 (0.01)	0.82 (0.00)	0.97 (0.01)	0.97 (0.01)	0.98 (0.00)	0.97 (0.01)
Positive predictive value	0.23 (0.02)	0.10 (0.01)	0.62 (0.05)	0.64 (0.05)	0.68 (0.07)	0.66 (0.06)
Negative predictive value	0.92 (0.00)	0.90 (0.01)	0.94 (0.01)	0.94 (0.01)	0.93 (0.01)	0.94 (0.01)
Area under the curve	0.72 (0.01)	0.52 (0.02)	0.92 (0.01)	0.91 (0.01)	0.92 (0.01)	0.92 (0.01)

Abbreviations: BDSI, Brief Dementia Screening Indicator; CAIDE, Cardiovascular Risk Factors, Aging and Incidence of Dementia; LR, logistic regression; RF, random forest; SVM, support vector machine; XGB, gradient-boosted trees.

^a^ Given values are for recommended thresholds.

^b^ Values are for a decision threshold of 0.5.

### Model Performance Across Dementia Subtypes

To assess the ML model performance in different dementia subtypes, we divided the population into 4 dementia subtypes: Alzheimer dementia (1285 participants), LBD (82 participants), vascular dementia (21 participants), and other dementia subtypes (180 participants). The LR model was best at identifying Alzheimer dementia and other subtypes, correctly classifying 589 participants (46%) with Alzheimer dementia and 99 participants (55%) with other subtypes. The SVM model performed best on participants with LBD, correctly classifying 40 participants (49%). All models correctly classified 7 participants (33%) with vascular dementia. Receiver operating characteristic curves demonstrate that all models performed approximately equally well on each subtype (eFigure 1 in the Supplement).

### Investigation of Minimum Number of Variables

**Figure 2.**
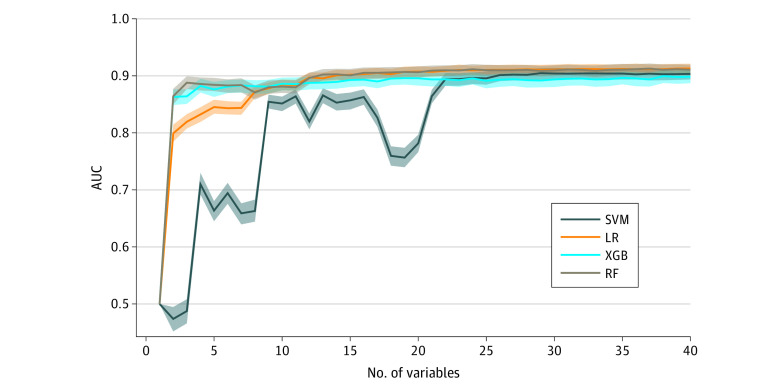
Area Under the Curve (AUC) vs the Number of Variables Used for Training for 4 Machine Learning Models Values were obtained by bootstrapping the validation set. Lines indicate the median; shaded regions, IQR; LR, logistic regression; RF, random forest; SVM, support vector machine; XGB, gradient-boosted trees.

One potential drawback of using an ML approach is the large number of variables involved. As the number of variables required by a model increases, implementation in a clinical setting becomes less practical and interpretability of the model is impaired. To assess how many variables each ML model required to achieve the equivalent predictive power to what we found using all 258 variables (Table 2), we evaluated how AUC varied with the number of variables included in the models. Specifically, we ranked the variables for each model by sorting them in descending order of importance (ie, the discriminatory power of each variable according to the algorithm; eMethods in the Supplement). We subsequently retrained each model with an increasing number of variables, starting with the most important. We found that all models required only 22 variables to achieve diagnostic performance statistically indistinguishable from their optimum mean performance (Figure 2; eFigure 2 in the Supplement). The synthetic variables, added to ensure the validity of variable importance assessment were not in the top 22 variables for any model, reflecting the fact that after full diagnostic performance was reached, there was little information to strongly determine the variable ranking.

### Identification of Key Risk Factors

Out of the 22 most important variables for each model, only 5 were common to all models (ie, clinical judgement of decline in memory, cognitive abilities, behavior, ability to manage affairs, or motor and movement changes; time to complete Trail Making Test Part B; CDR: orientation impairment; CDR: home and hobbies impairment; and level of independence). Of the remaining variables, there were 10 pairs that had a correlation greater than 0.7, indicating that they were similar variables (eTable 3 in the Supplement). Accounting for this correlation by interchanging variables that were highly correlated, we found that there were 6 highly predictive variables (clinical judgement of decline, time to complete Trail Making Test Part B, 3 components of the CDR [orientation, memory, and home and hobbies impairment], and level of independence) that were common to all ML models (eTable 4 in the Supplement). Training each model using only these variables, we found that for LR and XGB, there was no significant decrease in diagnostic performance: using this core set of 6 variables, these models had mean (SD) accuracy of 91% (0%) for LR and 91% (1%) for XGB and mean (SD) AUC of 0.89 (0.01) for LR and 0.89 (0.02) for XGB (eTable 5 in the Supplement).

### Diagnostic Stability

Of 1568 participants who received a diagnosis of dementia within 2 years, we identified 130 (8%) as experiencing reversion who were likely initially misdiagnosed and therefore mislabeled for ML purposes. We found that while reversions were only reported in 0.8% of participants, they accounted for 92 to 109 participants (7%-8%) of misclassified participants, with a small amount of variation between models (Table 3). The RF model had the highest diagnostic stability, correctly identifying 109 of 130 participants with reversion (84%) by classing them as predicted to be dementia free at 2 years. To investigate the diagnostic stability of ML models, we removed the participants with reversion during training (eMethods in the Supplement). After retraining the models without reversions, we found that RF identified 106 participants who experienced reversions (median [IQR], 82% [78%-82%]), SVM identified 93 participants who experienced reversions (median [IQR], 72% [69%-74%]), and LR and XGB both identified 92 participants who experienced reversions (median [IQR], 71% [68%-75%]). IQRs were obtained by bootstrapping participants who experienced reversion.

**Table 3.** Diagnostic Stability and Model Predictions Among Patients Who Were Initially Diagnosed With Dementia Within 2 Years of Their Baseline Assessment

Diagnosis status	Patients, No. (%)					
	BDSI	CAIDE	LR	SVM	RF	XGB
**Correctly classified**						
Consistently diagnosed, model predicted to develop dementia	536 (37.3)	243 (16.9)	694 (48.3)	689 (47.9)	477 (33.2)	666 (46.3)
Diagnosis reversed, model predicted to stay dementia-free^a^	91 (70.0)	97 (74.6)	92 (70.8)	93 (71.5)	109 (83.8)	98 (75.4)
**Misclassified**						
Consistently diagnosed, model predicted to stay dementia-free	902 (62.7)	1195 (83.1)	744 (51.7)	749 (52.1)	961 (66.8)	772 (53.7)
Diagnosis reversed, model predicted to develop dementia^a^	39 (30.0)	33 (25.4)	38 (29.2)	37 (28.5)	21 (16.2)	32 (24.6)

Abbreviations: BDSI, Brief Dementia Screening Indicator; CAIDE, Cardiovascular Risk Factors, Aging and Incidence of Dementia; LR, logistic regression; RF, random forest; SVM, support vector machine; XGB, gradient-boosted trees.

^a^ Patients considered as having their diagnosis reversed were initially diagnosed with dementia within 2 years of their baseline visit whose diagnosis was subsequently reversed to mild cognitive impairment or cognitively unimpaired within 2 years of further follow-up suggesting probable misdiagnosis.

To understand the difference between misclassified participants, participants with reversion, and participants who developed dementia without reversion, we analyzed the CDFs of classification scores obtained from each ML model. We found that the scores of misclassified participants, and specifically participants with reversion, were different from participants who developed dementia and those who did not (eFigure 3 in the Supplement). The CDFs of classification scores for participants who did not develop dementia fell to the far left of each plot, indicating that the ML models assigned these participants a low probability of developing dementia. Conversely, for participants who did develop dementia, the CDFs fell to the right of the plots: they were assigned a high probability of developing dementia. For all models, the distribution of scores for participants with reversion fell to the left of that for participants who did develop dementia, meaning that participants with reversion were assessed as having lower probability of developing dementia according to these models.

## Discussion

In this prognostic study, ML algorithms had superior prognostic accuracy compared with BDSI and CAIDE at predicting dementia incidence within 2 years of a patient’s first memory clinic assessment. Two of the ML algorithms assessed achieve an accuracy of 91% and AUC of 0.89 with only 6 key variables. Sensitivity analyses suggest that ML models could correctly classify a high proportion of participants who experienced reversion who were potentially misdiagnosed within 2 years of their initial visit. This study has several strengths, including the large sample of patients derived from multiple memory clinics across the United States, the wide range of ML techniques used, the benchmarking against existing risk models, and the exploration of diagnostic stability and probable misdiagnosis.

Prior studies into the use of ML for predicting dementia risk have focused on conversion from unimpaired cognition to Alzheimer dementia or MCI,^6,8^ or conversion from MCI to Alzheimer dementia.^5^ These approaches are less useful in a clinical setting, as they exclude other types of dementia^5,6,8^ or patients who are initially cognitively unimpaired.^5^ Data used in these studies included positron emission tomography scans,^5,8^ and cerebrospinal fluid biomarkers,^8^ which are not commonly available in a memory clinic setting. A study by Lin et al^6^ overcame this by using NACC data to find a set of 15 noninvasive clinical variables to assess risk of conversion from unimpaired cognition to MCI in a 4-year period. However, the construct of MCI remains somewhat controversial,^32^ and conversion rates between MCI and dementia are often low.^32,33^ Our ML models complement these analyses and have the advantage of incorporating only 6 key variables over a clinically relevant timescale and predicting the outcome of all-cause dementia.

Of the existing models investigated in our study, the CAIDE model was the least accurate in predicting dementia risk over 2 years, which is not surprising, given that it was developed to predict long-term dementia risk in middle-aged adults over a much longer follow-up period of 20 years. The BDSI performed better than the CAIDE, likely reflecting that it was designed for use in older adults over a more moderate follow-up period of 6 years. However, all ML models outperformed these existing models. Using all variables, XGB was the most powerful ML approach in predicting patients who were likely to be diagnosed with dementia within 2 years, suggesting that the way in which new decision trees are trained to correct the errors of the last tree results in a marginal performance gain. However, XGB also seemed to be the approach least able to identify participants who experienced reversion, ie, those who were initially diagnosed with dementia within 2 years and had that diagnosis reversed within 2 years of the initial diagnosis.

The performance of ML models can be considerably reduced by mislabeled training data.^34^ Counterintuitively, excluding mislabeled training data does not always improve the performance.^35^ As the level of noise in the training data increases, the value of excluding or reducing that noise decreases if the same noise is present in the validation data.^36^ Thus, filtering training data may even reduce performance in validation data, as found in this study. However, when the level of mislabeling is less than approximately 20% to 40%, removing mislabeled data can improve validation data accuracy, even if that incorporates mislabeled data.^35,37,38^ This illustrates the importance of investigating diagnostic stability in the training and validation data: even criterion standard data incorporates errors.

The observed rate of reversion (8%) was similar to that found in a 2019 study based on a different US population.^31^ In our study, the percentage of false positives was found to vary from 7% to 19%, depending on the cognitive assessment used. To our knowledge, this is the first analysis of potential misdiagnosis in the NACC UDS and suggests that using ML as a clinical decision-making aid has the potential to reduce the misdiagnosis of false positives by up to 84%. Given that patients who experience reversion are borderline in a diagnostic sense, from a clinical perspective, it may be sensible that they are followed anyway, given that there have been grounds for clinical concern. Thus, XGB may be the best model for a clinical decision-making aid. Alternatively, an ensemble approach that makes secondary predictions about probable diagnostic stability and the potential for misclassification may prove even more useful.

### Limitations

This study has several limitations. First, both CAIDE and BDSI were developed using different populations to the one used in this study. Not all variables used for the development of these models had an exact equivalent in the UDS which may have affected their performance in this data set. Second, the method used to impute the data may result in imputation error. Specifically, the imputation replaces all missing values with a numerical value, yet some values are missing owing to their relationship with another value; therefore, the fact that a value is missing is informative. However, while participants had a mean of 14% missing data, the 6 key variables identified were missing for a mean of 1% of participants. Third, although our study used a large sample of memory clinic attendees in the United States, making our results highly applicable to this setting, the extent to which these results will generalize to other populations is unknown.

## Conclusions

This prognostic study found that ML models outperformed existing dementia risk prediction models and may have the potential to improve the prediction of incident dementia over 2 years in memory clinics. Six key factors for dementia risk identified in this study may have the potential to improve clinical practice in memory clinics if incorporated into future clinical decision-making aids.

## Notes

Supplement. **eTable 1.** Total 258 Predictor Variables Used in Machine Learning Models
eMethods
**eTable 2.** Predictor Variables Used in Existing Models and the Equivalent Variables Within NACC UDS **eFigure 1.** Comparison of Receiver Operating Characteristic Curves for Machine Learning Models With the Validation Set Divided in 4 Dementia Subtypes **eFigure 2.** The 22 Most Important Variables for Machine Learning Models **eTable 3.** The 10 Pairs of Highly Correlated Variables **eTable 4.** The 6 Predictive Variables and Their Ranks After Substituting for Highly Correlated Variables **eTable 5.** Performance Measures for ML Models Using 6 Common Variables With a Threshold of 0.5 **eFigure 3.** Cumulative Distribution Functions of the Classification Scores From Machine Learning Models
eReferences

## References

[1] Hejl A, Høgh P, Waldemar G. Potentially reversible conditions in 1000 consecutive memory clinic patients. J Neurol Neurosurg Psychiatry. 2002;73(4):390-394. doi:10.1136/jnnp.73.4.390 12235305 PMC1738080

[2] Mitchell AJ, Shiri-Feshki M. Rate of progression of mild cognitive impairment to dementia—meta-analysis of 41 robust inception cohort studies. Acta Psychiatr Scand. 2009;119(4):252-265. doi:10.1111/j.1600-0447.2008.01326.x 19236314

[3] Barnes DE, Beiser AS, Lee A, . Development and validation of a brief dementia screening indicator for primary care. Alzheimers Dement. 2014;10(6):656-665.e1. doi:10.1016/j.jalz.2013.11.006 24491321 PMC4119094

[4] Kivipelto M, Ngandu T, Laatikainen T, Winblad B, Soininen H, Tuomilehto J. Risk score for the prediction of dementia risk in 20 years among middle aged people: a longitudinal, population-based study. Lancet Neurol. 2006;5(9):735-741. doi:10.1016/S1474-4422(06)70537-3 16914401

[5] Cui Y, Liu B, Luo S, ; Alzheimer’s Disease Neuroimaging Initiative. Identification of conversion from mild cognitive impairment to Alzheimer’s disease using multivariate predictors. PLoS One. 2011;6(7):e21896. doi:10.1371/journal.pone.0021896 21814561 PMC3140993

[6] Lin M, Gong P, Yang T, Ye J, Albin RL, Dodge HH. Big data analytical approaches to the NACC dataset: aiding preclinical trial enrichment. Alzheimer Dis Assoc Disord. 2018;32(1):18-27. doi:10.1097/WAD.0000000000000228 29227306 PMC5854492

[7] Park JH, Cho HE, Kim JH, . Machine learning prediction of incidence of Alzheimer’s disease using large-scale administrative health data. NPJ Digit Med. 2020;3(1):46. doi:10.1038/s41746-020-0256-0 32258428 PMC7099065

[8] Zhan Y, Chen K, Wu X, ; Alzheimer’s Disease Neuroimaging Initiative. Identification of conversion from normal elderly cognition to Alzheimer’s disease using multimodal support vector machine. J Alzheimers Dis. 2015;47(4):1057-1067. doi:10.3233/JAD-142820 26401783 PMC6287610

[9] Burgos N, Colliot O. Machine learning for classification and prediction of brain diseases: recent advances and upcoming challenges. Curr Opin Neurol. 2020;33(4):439-450. doi:10.1097/WCO.0000000000000838 32657885

[10] Beekly DL, Ramos EM, Lee WW, ; NIA Alzheimer’s Disease Centers. The National Alzheimer’s Coordinating Center (NACC) database: the Uniform Data Set. Alzheimer Dis Assoc Disord. 2007;21(3):249-258. doi:10.1097/WAD.0b013e318142774e 17804958

[11] National Institute on Aging. Alzheimer’s Disease Research Centers. Accessed May 21, 2021. https://www.nia.nih.gov/health/alzheimers-disease-research-centers

[12] Pfeffer RI, Kurosaki TT, Harrah CH Jr, Chance JM, Filos S. Measurement of functional activities in older adults in the community. J Gerontol. 1982;37(3):323-329. doi:10.1093/geronj/37.3.323 7069156

[13] Kaufer DI, Cummings JL, Ketchel P, . Validation of the NPI-Q, a brief clinical form of the Neuropsychiatric Inventory. J Neuropsychiatry Clin Neurosci. 2000;12(2):233-239. doi:10.1176/jnp.12.2.233 11001602

[14] Weintraub S, Salmon D, Mercaldo N, . The Alzheimer’s Disease Centers’ Uniform Data Set (UDS): the neuropsychologic test battery. Alzheimer Dis Assoc Disord. 2009;23(2):91-101. doi:10.1097/WAD.0b013e318191c7dd 19474567 PMC2743984

[15] Morris JC, Weintraub S, Chui HC, . The Uniform Data Set (UDS): clinical and cognitive variables and descriptive data from Alzheimer Disease Centers. Alzheimer Dis Assoc Disord. 2006;20(4):210-216. doi:10.1097/01.wad.0000213865.09806.92 17132964

[16] McKhann G, Drachman D, Folstein M, Katzman R, Price D, Stadlan EM. Clinical diagnosis of Alzheimer’s disease: report of the NINCDS-ADRDA Work Group under the auspices of Department of Health and Human Services Task Force on Alzheimer’s Disease. Neurology. 1984;34(7):939-944. doi:10.1212/WNL.34.7.939 6610841

[17] Román GC, Tatemichi TK, Erkinjuntti T, . Vascular dementia: diagnostic criteria for research studies: report of the NINDS-AIREN International Workshop. Neurology. 1993;43(2):250-260. doi:10.1212/wnl.43.2.250 8094895

[18] McKeith IG, Dickson DW, Lowe J, ; Consortium on DLB. Diagnosis and management of dementia with Lewy bodies: third report of the DLB Consortium. Neurology. 2005;65(12):1863-1872. doi:10.1212/01.wnl.0000187889.17253.b1 16237129

[19] Neary D, Snowden JS, Gustafson L, . Frontotemporal lobar degeneration: a consensus on clinical diagnostic criteria. Neurology. 1998;51(6):1546-1554. doi:10.1212/WNL.51.6.1546 9855500

[20] Martínez-Martín P, Gil-Nagel A, Gracia LM, Gómez JB, Martínez-Sarriés J, Bermejo F; The Cooperative Multicentric Group. Unified Parkinson’s disease rating scale characteristics and structure. Mov Disord. 1994;9(1):76-83. doi:10.1002/mds.870090112 8139608

[21] Morris JC. Clinical Dementia Rating: a reliable and valid diagnostic and staging measure for dementia of the Alzheimer type. Int Psychogeriatr. 1997;9(S1)(suppl 1):173-176. doi:10.1017/S1041610297004870 9447441

[22] Hastie T, Tibshirani R, Friedman J. The Elements of Statistical Learning: Data Mining, Inference, and Prediction. Springer Science & Business Media; 2009. doi:10.1007/978-0-387-84858-7

[23] Hosmer Jr DW, Lemeshow S, Sturdivant RX. Applied Logistic Regression. John Wiley & Sons; 2013. doi:10.1002/9781118548387

[24] Cortes C, Vapnik V. Support-vector networks. Mach Learn. 1995;20(3):273-97. doi:10.1007/BF00994018

[25] Breiman L. Random forests. Mach Learn. 2001;45(1):5-32. doi:10.1023/A:1010933404324

[26] Ho TK. Random decision forests. In: Proceedings of the 3rd International Conference on Document Analysis and Recognition. IEEE; 1995:278-282.

[27] Friedman JH. Stochastic gradient boosting. Computational Stat Data Analysis. 2002; 38(4):367-78. doi:10.1016/S0167-9473(01)00065-2

[28] Pedregosa F, Varoquaux G, Gramfort A, , Scikit-learn: machine learning in Python. J Mach Learn Res. 2011; 12:2825-2830.

[29] Krzanowski WJ, Hand DJ. ROC Curves for Continuous Data. CRC Press; 2009. doi:10.1201/9781439800225

[30] Fawcett, T. An introduction to ROC analysis. Pattern Recognition Lett. 2006;27(8):861-874. doi:10.1016/j.patrec.2005.10.010

[31] Ranson JM, Kuźma E, Hamilton W, Muniz-Terrera G, Langa KM, Llewellyn DJ. Predictors of dementia misclassification when using brief cognitive assessments. Neurol Clin Pract. 2019;9(2):109-117. doi:10.1212/CPJ.0000000000000566 31041124 PMC6461420

[32] Bruscoli M, Lovestone S. Is MCI really just early dementia: a systematic review of conversion studies. Int Psychogeriatr. 2004;16(2):129-140. doi:10.1017/S1041610204000092 15318760

[33] Farias ST, Mungas D, Reed BR, Harvey D, DeCarli C. Progression of mild cognitive impairment to dementia in clinic- vs community-based cohorts. Arch Neurol. 2009;66(9):1151-1157. doi:10.1001/archneurol.2009.106 19752306 PMC2863139

[34] Guan D, Yuan W, Ma T, Khattak AM, Chow F. Cost-sensitive elimination of mislabeled training data. Inf Sci. 2017; 402:170-81. doi:10.1016/j.ins.2017.03.034

[35] Brodley CE, Friedl MA. Identifying mislabeled training data. J Artif Intelligence Res. 1999;11:131-67. doi:10.1613/jair.606

[36] Quinlan JR. Induction of decision trees. Mach Learn. 1986;1(1):81-106. doi:10.1007/BF00116251

[37] Brodley CE, Friedl MA. Identifying and eliminating mislabeled training instances. In: AAAI ’96: Proceedings of the Thirteenth National Conference on Artificial Intelligence. AAAI; 1996:799-805.

[38] Brodley CE, Friedl MA. Improving automated land cover mapping by identifying and eliminating mislabeled observations from training data. In: IGARSS '96: 1996 International Geoscience and Remote Sensing Symposium. IEEE;1996:1379-1381. doi:10.1109/IGARSS.1996.516669

