# Supplementary material for: Performance of Machine Learning Algorithms for Predicting Progression to Dementia in Memory Clinic Patients
Source: JAMA Netw Open. 2021 Dec 16;4(12):e2136553. doi: 10.1001/jamanetworkopen.2021.36553 (PMC8678688; doi:10.1001/jamanetworkopen.2021.36553)
Supplement: Supplement. — eTable 1. Total 258 Predictor Variables Used in Machine Learning Models eMethods eTable 2. Predictor Variables Used in Existing Models and the Equivalent Variables Within NACC UDS eFigure 1. Comparison of Receiver Operating Characteristic Curves for Machine Learning Models With the Validation Set Divided in 4 Dementia Subtypes eFigure 2. The 22 Most Important Variables for Machine Learning Models eTable 3. The 10 Pairs of Highly Correlated Variables eTable 4. The 6 Predictive Variables and Their Ranks After Substituting for Highly Correlated Variables eTable 5. Performance Measures for ML Models Using 6 Common Variables With a Threshold of 0.5 eFigure 3. Cumulative Distribution Functions of the Classification Scores From Machine Learning Models eReferences [file jamanetwopen-e2136553-s001.pdf]

Supplemental Online Content

James C, Ranson JM, Everson R, Llewellyn DJ. Performance of machine learning algorithms for predicting progression to dementia in memory clinic patients. *JAMA Netw Open*. 2021;4(12):e2136553. doi:10.1001/jamanetworkopen.2021.36553

eTable 1. Total 258 Predictor Variables Used in Machine Learning Models
eMethods
eTable 2. Predictor Variables Used in Existing Models and the Equivalent Variables Within NACC UDS
eFigure 1. Comparison of Receiver Operating Characteristic Curves for Machine Learning Models With the Validation Set Divided in 4 Dementia Subtypes
eFigure 2. The 22 Most Important Variables for Machine Learning Models
eTable 3. The 10 Pairs of Highly Correlated Variables
eTable 4. The 6 Predictive Variables and Their Ranks After Substituting for Highly Correlated Variables
eTable 5. Performance Measures for ML Models Using 6 Common Variables With a Threshold of 0.5
eFigure 3. Cumulative Distribution Functions of the Classification Scores From Machine Learning Models
eReferences

This supplemental material has been provided by the authors to give readers additional information about their work.

© 2021 James C et al. *JAMA Network Open.*

eTable 1. Total 258 Predictor Variables Used in Machine Learning Models

NACCREAS	NACCEMD	BPSYS	GAIT	TAXES	COURSE
NACCREFR	NACCEPMD	BPDIAS	POSSTAB	SHOPPING	FRSTCHG
BIRTH_#MOS	NACCDBMD	HRATE	BRADYKIN	GAMES	MMSELOC
SEX	CVHATT	VISION	PDNORMAL	STOVE	MMSELAN
HISPANIC	CVAFIB	VISCORR	MEMORY	MEALPREP	MMSEORDA
HISPOR	CVANGIO	VISWCORR	ORIENT	EVENTS	MMSEORDA_PROB
PRIMLANG	CVBYPASS	HEARING	JUDGMENT	PAYATTN	MMSEORLO
EDUC	CVPACE	HEARAID	COMMUN	REMDATES	MMSEORLO_PROB
NACCLIVS	CVCHF	HEARWAID	HOMEHOBB	TRAVEL	NACCMMSE
INDEPEND	CVOTHR	NACCBMI	PERSCARE	NACCNREX	NACCMMSE_PROB
RESIDENC	CBSTROKE	ABRUPT	CDRSUM	FOCLDEF	NPSYCLOC
MARISTAT	NACCSTYR_#YRS	STEPWISE	CDRGLOB	GAITDIS	NPSYLAN
HANDED	CBTIA	SOMATIC	NPIQINF	EYEMOVE	LOGIMEM
NACCAGE	NACCTIYR_#YRS	EMOT	DEL_SEV	DECSUB	LOGIMEM_PROB
NACCNIHR	PD	HXHYPER	HALL_SEV	DECIN	DIGIF
INBIR_#MOS	PDYR_#YRS	HXSTROKE	AGIT_SEV	DECCLIN	DIGIF_PROB
INSEX	PDOTHR	FOCLSYM	DEPD_SEV	DECAGE	DIGIFLEN
INRELTO	PDOTHRYR_#YRS	FOCLSIGN	ANX_SEV	COGMEM	DIGIFLEN_PROB
INLIVWTH	SEIZURES	HACHIN	ELAT_SEV	COGJUDG	DIGIB
INVISITS	TRAUMBRF	SPEECH	APA_SEV	COGLANG	DIGIB_PROB
INCALLS	TRAUMEXT	FACEXP	DISN_SEV	COGVIS	DIGIBLEN
INRELY	TRAUMCHR	TRESTFAC	IRR_SEV	COGATTN	DIGIBLEN_PROB
NACCMOM	NCOTHR	TRESTRHD	MOT_SEV	COGOTHR	ANIMALS
NACCDAD	HYPERTEN	TRESTLHD	NITE_SEV	NACCCOGF	ANIMALS_PROB
NACCFAM	HYPERCHO	TRESTRFT	APP_SEV	COGMODE	VEG
ANYMEDS	DIABETES	TRESTLFT	NOGDS	BEAPATHY	VEG_PROB
NACCAMD	B12DEF	TRACTRHD	SATIS	BEDEP	TRAILA
NACCHTNC	THYROID	TRACTLHD	DROPACT	BEVHALL	TRAILA_PROB
NACCACEI	INCONTU	RIGDNECK	EMPTY	BEAHALL	TRAILB
NACCAAAS	INCONTF	RIGDUPRT	BORED	BEDEL	TRAILB_PROB
NACCBETA	DEP2YRS	RIGDUPLF	SPIRITS	BEDISIN	WAIS
NACCCCBS	DEPOTHR	RIGDLORT	AFRAID	BEIRRIT	WAIS_PROB
NACCDIUR	ALCOHOL	RIGDLOLF	HAPPY	BEAGIT	MEMUNITS
NACCVASD	TOBAC30	TAPSRT	HELPLESS	BEPERCH	MEMUNITS_PROB
NACCANGI	TOBAC100	TAPSLF	STAYHOME	BEOTHR	MEMTIME
NACCAHTN	SMOKYRS	HANDMOVR	MEMPROB	NACCBEHF	BOSTON
NACCLIPL	PACKSPER	HANDMOVL	WONDRFUL	BEMODE	BOSTON_PROB
NACCNSD	QUITSMOK	HANDALTR	WRTHLESS	MOGAIT	COGSTAT
NACCAC	ABUSOTHR	HANDALTL	ENERGY	MOFALLS	NACCC1
NACCADEP	PSYCDIS	LEGRT	HOPELESS	MOTREM	RANDVAR_0
NACCAPSY	NACCTBI	LEGLF	BETTER	MOSLOW	RANDVAR_1

© 2021 James C et al. *JAMA Network Open.*

NACCAANX	HEIGHT	ARISING	NACCGDS	NACCMOTF	RANDVAR_2
NACCPDMD	WEIGHT	POSTURE	BILLS	MOMODE	RANDVAR_3

eMethods

Machine learning models

Logistic Regression (LR) is a probabilistic model, meaning it assigns a class probability to each participant.[1] Probabilities are calculated using a logistic function. This function maps a linear combination of the variables of each participant to a value between 0 and 1, which may be viewed as a class probability. If the class probability is greater than a given "decision threshold", the participant is classified as belonging to class 1 (Dementia). For probabilities less than the threshold, they are placed in class 0 (No Dementia). The decision threshold may be varied to adjust the balance between the sensitivity and specificity of the resulting classifier, as explored subsequently.
In contrast to LR, Support Vector Machine (SVM) is a non-probabilistic binary classifier.[2] During training, an SVM classifier finds a boundary, or hyper-plane, that spatially separates the classes. The class to which a point is assigned is determined by which side of the hyper-plane it lies. The separating hyper-plane is chosen to be that which gives the largest margin, or largest separation, between the classes. Similar to LR, a score approximating the probability of class membership can be derived as a function of the distance of the point from the hyperplane. Using a linear SVM allowed for variable importance to be evaluated.
Random Forest (RF) and Gradient-Boosted Trees (XGB) are examples of ensemble learning algorithms, where the underlying algorithm is a decision tree. During training a RF will select a random sample of the training data with replacement and fit a decision tree to this sample.[3,4] This process is repeated many times, the exact number being a parameter of the algorithm, to create an ensemble (or forest) of decision trees. XGB differs from RF by training decision trees sequentially such that each new tree is trained to correct the errors from the previously trained tree.[5] At validation stage, the probability of a participant belonging to either class is determined by averaging over the outcomes obtained from applying each individual decision tree to the participant.
To apply the machine learning algorithms to our data, we used one-hot encoding of categorical variables, creating a new binary variable for each of the categories. We scaled the data such that each variable had a mean of zero and variance of one.

To perform the 5-fold cross validation we used the 'StratifiedKFold' function in sci-kit learn,[6] carrying out a parameter search for the models within each fold, using 'GridSearchCV' which performs an exhaustive search of a parameter grid for each model. The best set of parameters were used to specify each model, for each fold, resulting in up to 20 different models with different parameters. All code used for the modelling process is available online.[7]

Model evaluation

Performance measures were obtained by bootstrapping the data. Specifically, we selected a random sample of 1000 patients, with replacement, and calculated performance measures in this sample using the predicted classes and class probabilities obtained during 5-fold cross validation. The sampling was repeated 100 times to obtain a distribution of values for each measure. The standard deviation of the distribution of values of a measure is quoted as the error.

Variable importance

To assess the variable importance for each model we used the coef (LR and SVM) and feature importances (RF and XGB) functionality of scikit-learn.[6] For LR and SVM, the importance of a variable is determined by the magnitude of its coefficient when the model is fit to the training data; the larger the magnitude of the coefficient the more important a variable is to the prediction. For RF and XGB, variable importance is determined by the Gini importance.[8] Specifically, for each tree a variable's importance is the total decrease in impurity that occurs when the variable is used to split a node, weighted by the number of samples the node splits. The Gini importance is calculated for each tree and then averaged to give a final variable importance.

During the 5-fold cross validation, we determined variable importance for each fold. The final importance of each variable was calculated by averaging over the folds.

© 2021 James C et al. *JAMA Network Open.*

Diagnostic Stability

We define *reversion* as when a participant who was diagnosed with dementia up to 2 years after their first memory clinic visit subsequently receives a diagnosis of no dementia (either MCI or unimpaired cognition) within 2 years of their initial dementia diagnosis.

To investigate the classification accuracy of ML models in participants with reversion, we removed all such participants from the training data in each fold. This is justified by the definition of a reversion; in the data these participants are labelled as 1 (having incident dementia), yet when their subsequent diagnosis of no dementia is taken into account this diagnosis of dementia, and therefore their label, is incorrect. By removing participants with reversion from the training data we ensured that the ML models were trained on, to our knowledge, correctly labelled data only.

We subsequently re-trained the ML models to perform the same classification task, identify whether a participant would develop dementia within 2 years of their baseline assessment, without reversions in the training data. We assessed each model's ability to identify participants with reversion by looking at the labels assigned to these participants. If a participant with a reversion is classified as dementia free (class 0), they are identified; the ML model has labelled them correctly rather than misdiagnosing them, as they have been in the data.

© 2021 James C et al. *JAMA Network Open.*

eTable 2. Predictor Variables Used in Existing Models and the Equivalent Variables Within NACC UDS

Predictor	BDSI	CAIDE	UDS Variables
**Age**	Y	Y	NACCAGE
**Sex**		Y	SEX
**Education**	Y	Y	EDUC
**Activities**	Y		TAXES, BILLS, REMDATES
**Blood Pressure**		Y	BPSYS
**BMI**	Y	Y	NACCBMI
**Cholesterol**		Y	HYPERCHO
**Depression**	Y		NACCADEP, APA, APATHY
**Diabetes**	Y		DIABTYPE
**Stroke**	Y		HXSTROKE, CBSTROKE
**Physical Activity**		Y	STAYHOME

© 2021 James C et al. *JAMA Network Open.*

eFigure 1. Comparison of Receiver Operating Characteristic Curves for Machine Learning Models With the Validation Set Divided in 4 Dementia Subtypes

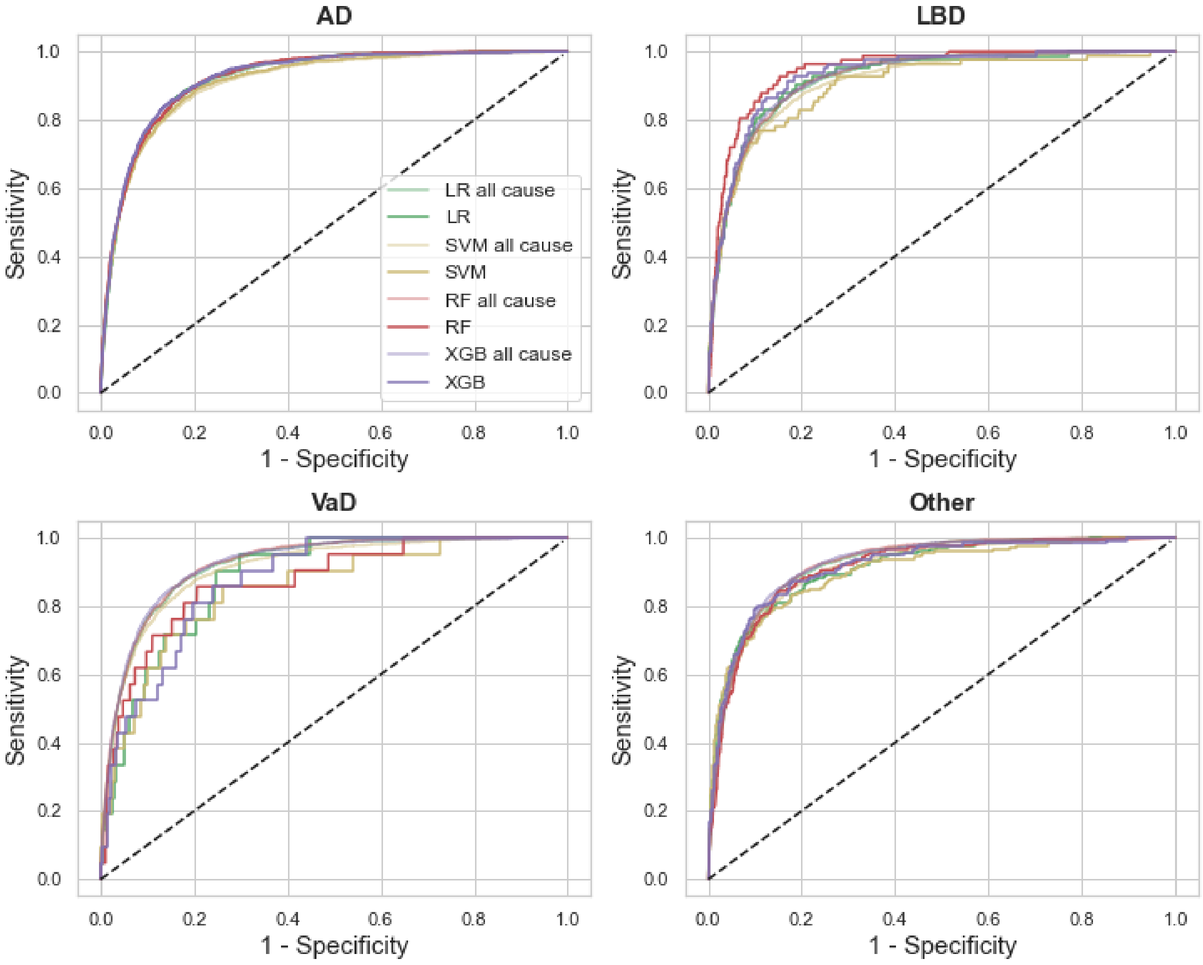

Notes: 'all cause' denotes Receiver Operating Characteristic curves for the full undivided validation set. Abbreviations: RF = Random Forest, LR = Logistic Regression, SVM = Support Vector Machine, XGB = Gradient-boosted Trees, AD = Alzheimer's Dementia, LDB = Dementia with Lewy Bodies, VaD = Vascular Dementia.

© 2021 James C et al. *JAMA Network Open.*

eFigure 2. The 22 Most Important Variables for Machine Learning Models

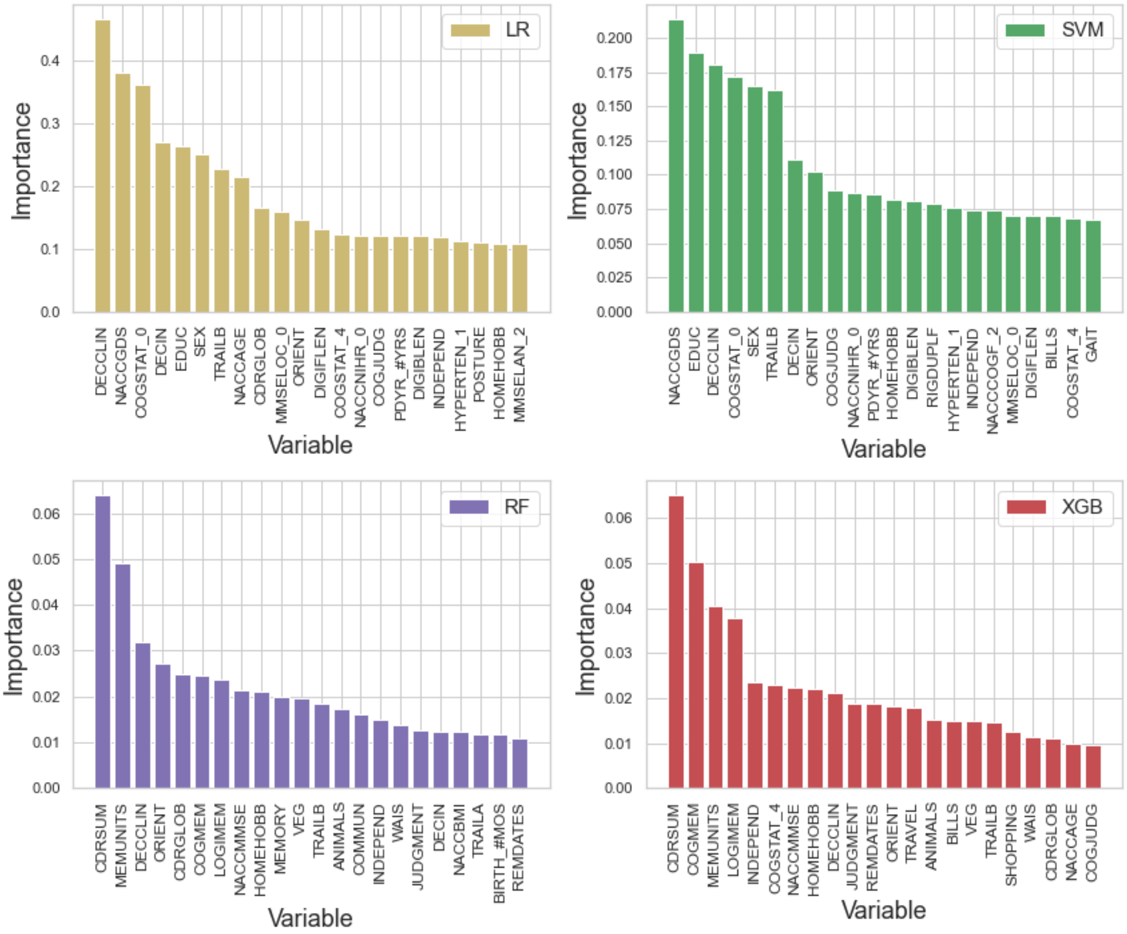

Abbreviations: RF = Random Forest, LR = Logistic Regression, SVM = Support Vector Machine, XGB = Gradient-boosted Trees.

© 2021 James C et al. *JAMA Network Open.*

eTable 3. The 10 Pairs of Highly Correlated Variables

VARIABLE 1	VARIABLE 2	CORRELATION
CDRSUM	COMMUN	0.79
CDRSUM	JUDGMENT	0.82
CDRSUM	CDRGLOB	0.76
CDRSUM	MEMORY	0.80
MEMORY	COGMEM	0.81
MEMORY	CDRGLOB	0.91
COGMEM	CDRGLOB	0.82
COGMEM	DECIN	0.71
JUDGEMENT	COGJUDG	0.74
MEMUNTIS	LOGIMEM	0.84

© 2021 James C et al. *JAMA Network Open.*

eTable 4: The 6 Predictive Variables and Their Ranks After Substituting for Highly Correlated Variables

**Variable**	**Description**	**LR rank**	**SVM rank**	**RF rank**	**XGB rank**
DECCLIN	Clinician believes there is a meaningful decline in memory, non-memory cognitive abilities, behaviour, ability to manage his/her affairs, or there are motor/movement changes	1	3	3	9
TRAILB	Trail Making Test Part b — Total number of seconds to complete	7	6	12	17
ORIENT	Orientation	11	8	4	12
MEMORY	Memory	4	7	1	1
HOMEHOBB	Home and hobbies	21	12	9	8
INDEPEND	Level of independence	18	16	15	5

Notes: Colours correspond to the following categories: Green, Neuropsychological Battery Summary scores; Yellow, Clinical judgement of symptoms; Blue, Clinical dementia rating; Orange, Subject demographics. Abbreviations: RF = Random Forest, LR = Logistic Regression, SVM = Support Vector Machine, XGB = Gradient-boosted Trees.

© 2021 James C et al. *JAMA Network Open.*

eTable 5. Performance Measures for ML Models Using 6 Common Variables With a Threshold of 0.5

Performance Measures[a]	LR	SVM	RF	XGB
**Accuracy**	0.91 (0.00)	0.90 (0.01)	0.90 (0.01)	0.91 (0.01)
**Sensitivity**	0.27 (0.04)	0.00 (0.00)	0.26 (0.04)	0.28 (0.04)
**Specificity**	0.98 (0.01)	1.00 (0.00)	0.97 (0.01)	0.98 (0.01)
**Positive Predictive Value**	0.62 (0.08)	0.00 (0.00)	0.55 (0.07)	0.59 (0.08)
**Negative Predictive Value**	0.92 (0.01)	0.90 (0.01)	0.92 (0.01)	0.92 (0.01)
**AUC[b]**	0.89 (0.01)	0.78 (0.02)	0.89 (0.02)	0.89 (0.02)

Notes: [a]Values represent mean (standard deviation) [b]AUC independent of threshold. Abbreviations: RF = Random Forest, LR = Logistic Regression, SVM = Support Vector Machine, XGB = Gradient-boosted Trees.

© 2021 James C et al. *JAMA Network Open.*

eFigure 3. Cumulative Distribution Functions of the Classification Scores From Machine Learning Models

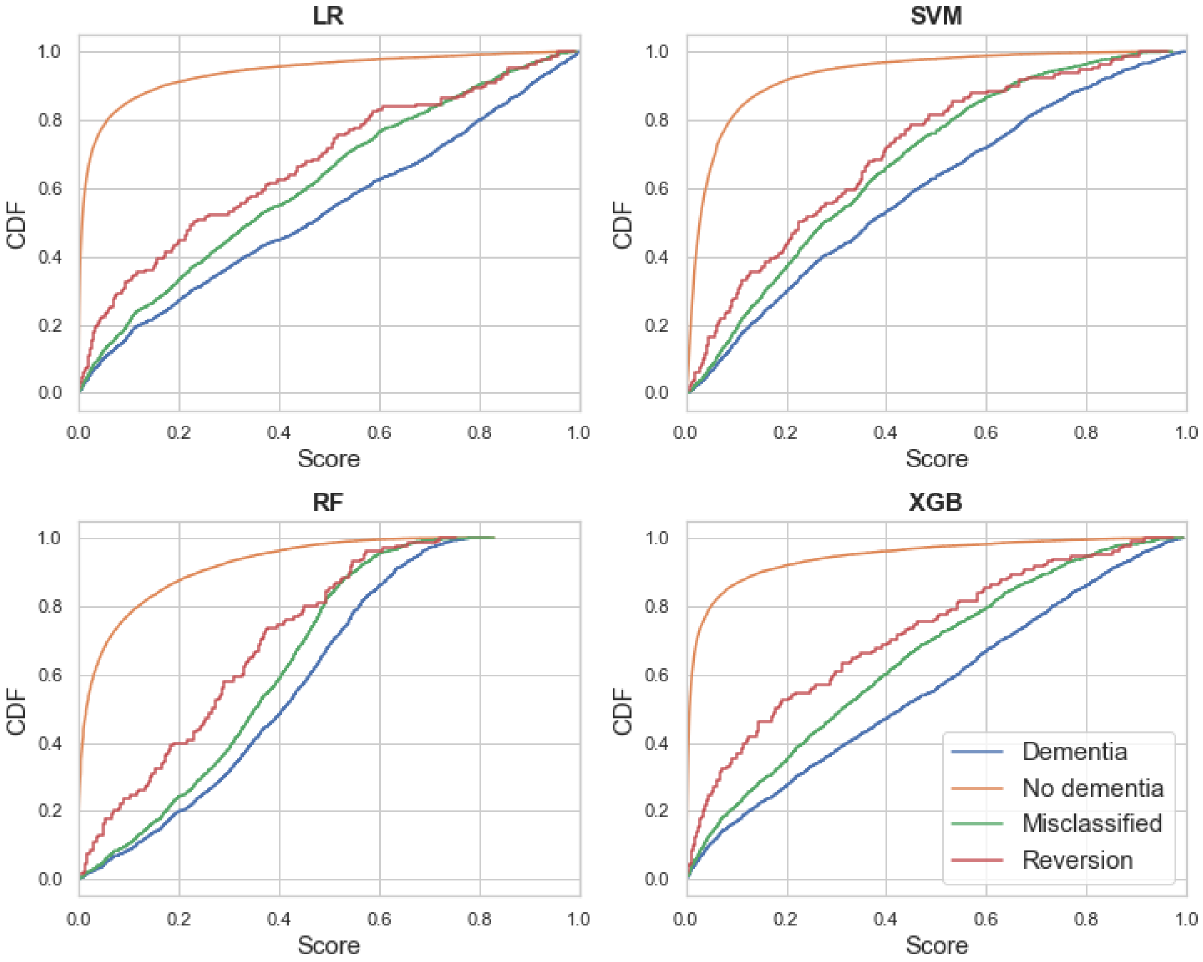

Abbreviations: RF = Random Forest, LR = Logistic Regression, SVM = Support Vector Machine, XGB = Gradient-boosted Trees.

© 2021 James C et al. *JAMA Network Open.*

eReferences

1. Hosmer Jr DW, Lemeshow S, Sturdivant RX. Applied logistic regression. John Wiley & Sons, 2013.
2. Cortes C, Vapnik V. Support-vector networks. Machine learning. 1995; 20(3):273-97.
3. Breiman L. Random forests. Machine learning. 2001 Oct;45(1):5-32.
4. Ho TK. Random decision forests. In Proceedings of 3rd international conference on document analysis and recognition. IEEE. 1995; 1:278-282.
5. Friedman JH. Stochastic gradient boosting. Computational statistics & data analysis. 2002; 38(4):367-78.
6. Pedregosa F, Varoquaux G, Gramfort A, *et al.*, Scikit-learn: Machine Learning in Python. J Mach Learn Res. 2011; 12:2825-2830.
7. https://github.com/charlotte-james/ML-dementia-progression
8. Friedman J, Hastie T, Tibshirani R. The elements of statistical learning. New York: Springer series in statistics; 2001.

© 2021 James C et al. *JAMA Network Open.*
